# Genetic modifications associated with sustainability aspects for sustainable developments

**DOI:** 10.1080/21655979.2022.2061146

**Published:** 2022-04-07

**Authors:** Pooja Sharma, Surendra Pratap Singh, Hafiz M.N. Iqbal, Roberto Parra-Saldivar, Sunita Varjani, Yen Wah Tong

**Affiliations:** a Environmental Research Institute, National University of Singapore, Singapore; b Energy and Environmental Sustainability for Megacities (E2S2) Phase II, Campus for Research Excellence and Technological Enterprise (CREATE), Singapore; c Plant Molecular Biology Laboratory, Department of Botany, D.A.V. College, Chhatrapati Shahu Ji Maharaj University, Kanpur, India; d Tecnologico de Monterrey, School of Engineering and Sciences, Monterrey, Mexico; e FEMSA, Tecnológico de MonterreyEscuela de Ingeniería y Ciencias- Centro de Biotecnología-, Monterrey, Mexico; f Gujarat Pollution Control Board, Gandhinagar, India; g Department of Chemical and Biomolecular Engineering, National University of Singapore, Singapore

**Keywords:** Transgenic crops, environment, biodiversity, sustainability, biotechnology

## Abstract

Sustainable development serves as the foundation for a range of international and national policymaking. Traditional breeding methods have been used to modify plant genomes and production. Genetic engineering is the practice of assisting agricultural systems in adapting to rapidly changing global growth by hastening the breeding of new varieties. On the other hand, the development of genetic engineering has enabled more precise control over the genomic alterations made in recent decades. Genetic changes from one species can now be introduced into a completely unrelated species, increasing agricultural output or making certain elements easier to manufacture. Harvest plants and soil microorganisms are just a few of the more well-known genetically modified creatures. Researchers assess current studies and illustrate the possibility of genetically modified organisms (GMOs) from the perspectives of various stakeholders. GMOs increase yields, reduce costs, and reduce agriculture’s terrestrial and ecological footprint. Modern technology benefits innovators, farmers, and consumers alike. Agricultural biotechnology has numerous applications, each with its own set of potential consequences. This will be able to reach its full potential if more people have access to technology and excessive regulation is avoided. This paper covers the regulations for genetically modified crops (GMCs) as well as the economic implications. It also includes sections on biodiversity and environmental impact, as well as GMCs applications. This recounts biotechnological interventions for long-term sustainability in the field of GMCs, as well as the challenges and opportunities in this field of research.

**Abbreviations:** GMCs-Genetically modified crops; GMOs- Genetically modified organisms; GE- Genetic engineering; Bt- *Bacillus thuringiensis*NIH- National Institutes of Health; FDA- Food and Drug Administration; HGT- Horizontal gene transfer; GM- Genetically modified; rDNA- Ribosomal deoxyribonucleic acid; USDA- United States Department of Agriculture; NIH- National Institutes of Health

## Graphical Abstract

**Figure:**
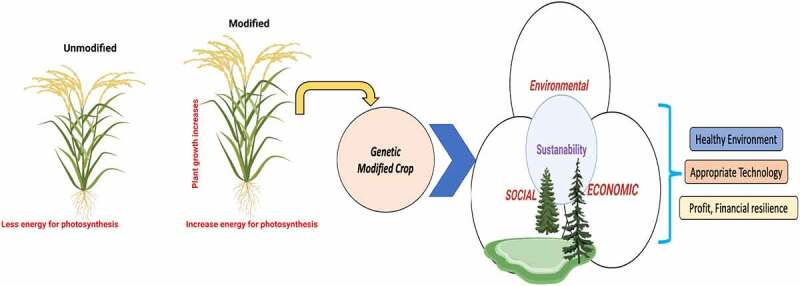

## 1. Introduction

Food is one of the most basic human needs; we eat to survive, and most people are fortunate to have at least one meal per day. Dining, regardless of our customs and culture, continues to be an important part of various celebrations around the world within and among friends and family [1,2]. Climate change adaptability, environmental sustainability, and food security are just a few of the primary concerns that genetic engineering (GE) can address. This equips farmers with new capabilities and implements to boost productivity, decrease environmental impact, encourage expanding peoples in emerging states, and encourage marginalized people. Different ideological groups, big corporations, and governments have voiced strong opposition to genetic engineering in agriculture: At the same time, different ideological groups, big corporations, and governments have voiced strong opposition to genetic engineering in agriculture. There are numerous published GE strategies for engineering disease resistance, and ongoing research and expanding genetic resources are likely to result in new approaches (Cochrane, [3]2010). Moreover, various applications are possible within the majority of those strategies. Taken together, these findings imply that GE opens up a vast pool of genetic possibilities for future generations. This will enable disease resistance breeding to remain highly dynamic in the face of pathogen adaptation to virulence on resistant cultivars. Because of the rapid growth of the worldwide people, climate change, food security, and the depletion of nonrenewable resources, the economy is becoming increasingly important as a tool for sustainable and inclusive employment generation [4–8]. The advancement of the bioeconomy necessitates public acceptance, particularly in the use of genetic engineering (GE) in agriculture and the marketing of genetically modified (GM) foods, which have a high economic value [9].

**Figure 1.**
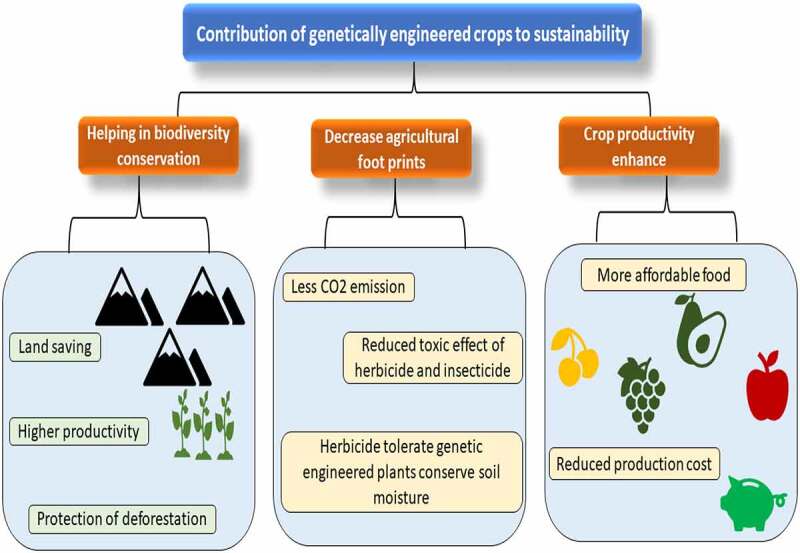
Contribution of genetically engineered crops for sustainability.

In 1946 scientists reported that DNA can be passed from one individual to another [10]. This is now known that various methods for DNA transfer exist and that they appear often in natural surroundings; for example, antibiotic resistance in harmful bacteria is a significant mechanism for this. In 1983, an antibiotic-resistant tobacco plant was used to create the first GM plant. China was the first country to commercialize a transgenic crop with the release of virus-resistant tobacco in the early 1990s. The Food and Drug Administration (FDA) approved the transgenic ‘Flavour Saver tomato’ for distribution in the United States in 1994. The tomato was able to postpone ripening after being picked as a result of the alteration. Few transgenic crops were approved for commercialization in 1995. Canola with a modified oil composition (Calgene), soybeans resistant to the herbicide glyphosate (Monsanto), Bt cotton (Monsanto), corn/maize (Ciba-Geigy), *Bacillus thuringiensis* (Bt) cotton resistant to the herbicide bromoxynil (Calgene), *Bacillus thuringiensis* (Bt) potatoes (Monsanto), and virus-resistant squash (Asgrow), and tomatoes late-maturing [10]. Until 1996, 35 approvals were granted in six countries and the European Union for the cultivation of eight transgenic crops and one flower crop of carnations with eight different traits (James et al., [11]1996). In the development of genetically modified crops, the United States leads a group of countries as of 2011. There are now a variety of food species that have been genetically engineered. Cotton, eggplant, soybeans, carrots, potatoes, canola, strawberries, corn, lettuce, tomatoes, cantaloupe, and other foods are accessible on the market. Vaccines and medicines, meals and food additives feed and fibers are among the GM items now in development. One of the most difficult parts of the procedure is finding genes for critical features like insect resistance or required nutrients. Crops that used this technique accounted for 48% of global plantings of these four crops in 2018. In addition, small areas of GM apples (the United States since 2016), papaya (China and United States since 1999 since 2008), brinjal (Bangladesh 2015), squash (the United States since 2004), sugar beet (the United States and Canada since 2008), alfalfa (the United States initially in 2005–2007 and then from 2011), and potatoes (the United States since 2015) [12]. This evaluation will also examine certain important concerns concerning GM foods and recombinant technology’s safety, environmental and ecological issues, and health risks. The contribution of genetically engineered crops to sustainability has been provided in Figure 1.

## 2. International regulations for genetically modified crops

The first dispute regarding the risks of GMO exposure to humans emerged in 1971 mutual abdominal bacterium, *E. coli*, was tainted with DNA [13]. Persons working in a research laboratory with GMOs, as well as neighboring people, were first concerned about safety concerns [14]. Several ethical concerns have been raised about horizontal gene transfer (HGT) from GMOs, including perceived threats to the integrity and intrinsic value of the organisms involved, the concept of natural order and species integrity, and the integrity of the ecosystems in which the genetically modified organism appears (Gene Technology Ethics Committee, ‘Working paper: ethical issues arising from trans-species gene transfer,’ 2006, http://www.ogtr.gov.au). Furthermore, apprehensions that recombinant creatures could be employed as weapons sparked further debate. The rising discussion, which began among scientists but soon moved to the general public, led to the formation of the recombinant DNA Advisory Committee by the National Institutes of Health (NIH) in 1974 to begin to address some of these challenges. When deliberate releases of GMOs into the environment began in the 1980s, the United States had very few laws in place. The industry was free to follow the NIH recommendations if they so desired. Throughout the 1980s, transgenic plants were used for the production of novel drugs was becoming a valuable enterprise, and individual corporations, organizations, and entire governments started to understand biotechnology as a profitable income of production currency [13]. The worldwide commercial of biotech crops has spawned fresh discussions about the potential of live creatures, the dangers of recombinant protein experience, privacy difficulties, scientists’ morality, and reliability, and the character of government in science guidelines. The worldwide commercialization of biotech products has spawned fresh debates about the patentability of live organisms, the dangers of recombinant protein exposure, confidentiality difficulties, scientists’ morality and credibility, the role of government in science regulation, and other topics. The congressional office of technology Assessment projects was created in the United States, and they were then replicated worldwide as a top-down approach to advise politicians by anticipating the general implications of GMOs. Risk evaluations are completed according to this document. Because the case-by-case method to hazard valuation for hereditarily altered goods has gained widespread acceptance; nonetheless, the United States has traditionally followed a product-based approach to evaluation, whilst Europe has taken a more process-based method [13]. Since many countries lacked comprehensive regulation in the former, administrations around the world are now responding to community pressure by enacting stronger testing and labeling standards for genetically engineered plants.

Since genetically engineered foods have become one of the most debated themes in past years. Several European environmental organizations, non-governmental organizations, and community attention assemblies have been disapproving of GM foods for years. Besides, current contentious research on the effects of genetically modified foods has brought genetic manufacturing to the public’s attention for sustainable development [15]. In general, the impression of releasing GM food for hominid feeding is not received in Europe due to health concerns [16]. Around are still no conclusive investigation findings indicating that GM foods are harmful to human health, avoiding them is more or less beneficial. Moreover, as the use of biofuels as an alternative source of energy becomes more popular, genetic engineering will become more important for financial reasons. As public concern about genetically modified foods grows, various governments around the world employ a variety of strategies to address the issue. GMOs rules have been developed, most of which are nation detailed. The European parliament and council, have enacted laws on genetically modified foods to safeguard citizens’ fitness and happiness, as well as European social and economic interests [17]. The EU laws distinguish between GM feed and GM food, and they also specify in what way GM crops must be considered in footings of the number of alterations they contain. Lower criteria should be possible, especially for foods and feed containing developments in research and expertise. The European GM food rules, in my opinion, are the strictest in the world, and it is unclear whether there is any room for GM products because of the restrictions’ complexity in understanding and application. “Nonetheless, the EU GMOs regulations could be summarized as follows: lay down community events for the authorization and management of genetically modified food and feed; and lay down provisions for a high level of defense of human, animal and welfare, the atmosphere, and consumer benefits concerning genetically modified food, simultaneously maintaining the internal market’s proper operation. The US Food and Drug Administration (FDA) evaluates if the plant is safe to eat; the US Environmental Protection Agency (EPA) determines if GM plants were environmentally safe, and the US Department of Agriculture (USDA) determines whether the growing facilities are safe (Pelletier, 2005). Many divisions within the USDA are responsible for evaluating GM foods. The Animal Health and Plant Inspection Service (APHIS) conducts field tests and issues permit to grow GM crops, the Agricultural Research Service conducts in-house GM food research, and the USDA risk assessment program is overseen by the Cooperative State Research, Education, and Extension Service [18].

**Figure 2.**
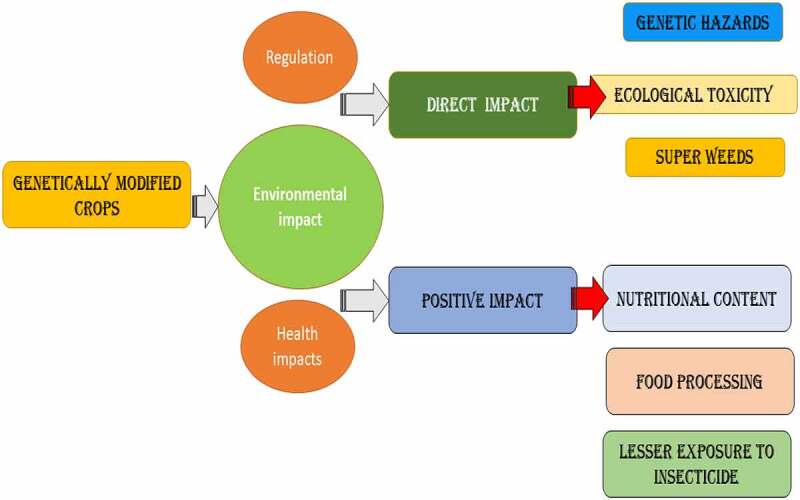
Regulation, environment and health impacts of GMs crops.

It means that to continue with GM food, a mixture of regulations from all three bodies must be obeyed. Nonetheless, it is claimed that up to 70% of processed foods on supermarket shelves in the United States include genetically altered components, ranging from soda to soup, and crackers to sauces. Furthermore, up to 85% of maize, 91% of soybeans, and 88% of cotton in the United States are genetically modified [18]. In several impoverished nations, where there is normally a season of plenty and a period of hunger owing to seasonal variations, GM food is less a problem since the drive is to feed the hungry people. Even if about of them have GMOs guidelines in place, their norms and laws are useless when food help is supplied to their country in the event of a tragedy. It’s appropriate because the main goal is to save lives before considering any other factors. Plants can withstand severe issues and, as a result, have adapted to changing environmental conditions by developing genes that are resistant to various toxins. It is proven by the fact that deviations in plants as a result of genetic alteration inbreeding were previously believed to be typically safe. However, in the early 1970s, when rDNA (ribosomal deoxyribonucleic acid) technology was introduced, Cohen and Boyer were able to successfully join two separate bits of DNA [19–21]. The benefits of genetic engineering in crop breeding were not recognized by the scientific community, but the risks connected with these approaches were [19]. Plant breeding in agriculture, in particular, has benefited from rapid and significant advances in research over the last century. Global scenario of health impacts of GMs crops with regulation concern has been provided in Figure 2.

## 3. Economic consequences

An additional concern about GMOs is firms may claim possession of the creatures they produce and refuse to segment them with the public at a sensible price. Whether these privileges are factual, this has contended that genetically modified crops determination harm the budget and the ecosystem since monoculture practices by large-scale farm production centers will trump the diversity contributed by small farmers who cannot access the innovation. Furthermore, according to a recent meta-analysis of 15 research, two-thirds of the assistances of first-generation genetically modified crops are distributed downstream, whereas only one-third is distributed upstream [22,23]. As a result, an indication from first-generation genetically modified crops refutes the assumption that private corporations will not part ownership of GMOs. Genetic modification application for abiotic stress, disease tolerance, and genetic modification applications for nutrients has been provided in Table 1.

**Table 1.** Genetic modification application for abiotic stress

S. No.	Trait groups	Targets	Plant species	Approaches	References
1.	Abiotic stress	Drought tolerance	Maize	CRISPR/Cas9	[24]
2.	Abiotic stress	Thermotolerance	Cattle	CRISPR/Cas9	25
]3.	Abiotic stress	Drought tolerance	Rice	CRISPR/Cas9, CRISPR/Cpf1	26,27
4.	Abiotic stress	Early flowering	Rice	CRISPR/Cas9	28
5.	Abiotic stress	Salt tolerance	Rice	CRISPR/Cas9	29
6.	Abiotic stress	Semi-dwarfed	Banana	CRISPR/Cas9	30

## 4. Application of GMCs

New approaches to recover the agronomic presentation of harvests for feed, food, and dispensation have been created since the introduction of transgenic technologies. Nevertheless, by using transgenic technology to express foreign genes in plants, large-scale manufacturing of commercially useful industrial or pharmaceutical items has become possible. There are several worries around the environmental impact of GMC, given their high acceptance rates and prospects [31,32]. Because of the “potential for contamination of the food chains and environment, substantial consideration has been given to how such particles generate might be properly remote and confined.One logical step after producing a transgenic plant is to assess its possible environmental advantages and dangers, which must be associated with those created by old-style agriculture [33,34]. The precautionary approach to GM plant risk management may necessitate the monitoring of substantial weed and wild inhabitants that could be impacted by transgene release. To appropriately identify hazards and keep a lookout for future ones, any legal framework must have effective risk assessment and monitoring systems in place. To ensure the safe use of combinational and encourage positive overall development, several organizations in various countries control the release of GM organisms or design suggestions for the suitable application of recombinant organisms in agro-industries. Therefore, think it is crucial to establish an internationally accepted strategy for the safety assessment of recombinant DNA organisms within the next few years [35–42]. Co-transformation, particle bombardment or biolistic, site-specific recombinase-mediated marker deletion, transposon-based expulsion systems, and intrachromosomal recombination-based excision are all methods for creating marker-free transgenic plants [43,44]. Golden rice (in China, Philippines, and India), Potato (in North America), cassava (in Brazil), tomato, sweet potato, maize, broccoli, mustard oil (in India), and apple are some of the bioengineered agricultural commodities (in New Zealand) [45–47]. Genetic modification applications for disease tolerance and genetic modification applications for nutrients have been provided in Tables 2 and 3.

**Table 2.** Genetic modification applications for disease tolerance

Trait groups	Targets	Plant species	Approaches	References
Broad-spectrum	Disease	Barley	CRISPR/Cas9	48
Resistance to phytophthora	Disease	Cacao	CRISPR/Cas9	49
Potato virus Y	Disease	Potato	CRISPR/Cas9	50,51
Bacterial blight resistance	Disease	Rice	CRISPR/Cas9	52, 53
Bacterial blight	Disease	Rice	TALENs	54
Bacterial blight	Disease	Rice	CRISPR/Cas9	55
Powdery mildew	Disease	Wheat	CRISPR/Cas9; TALENS	56
Mastitis	Disease	Cattle	ZFN	57, 58
Bacterial speck	Disease	Tomato	CRISPR/Cas9	59,60
Banana streak	Disease	Banana	CRISPR/Cas9	61
Broad-spectrum	Disease	Barley	CRISPR/Cas9	48
Resistance to phytopthora	Disease	Cacao	CRISPR/Cas9	49, 62

**Table 3.** Genetic modification applications for nutrients

Trait group	Targets	Plant species	Approaches	References
Nutrition	Reduced starch	Cassava	CRISPR/Cas9	63, 64
	Reduced phytate levels	Maize	ZFN	65
	Reduced phytic acid	Maize	TALENs, CRISPR/Cas9	66
	Increased oleic acid content	Peanut	TALENs	67
	Reduced starch	Potato	CRISPR/Cas9	68–72
	Prevented cadmium uptake	Rice	CRISPR/Cas9	73
	Increased carotenoids	Rice	CRISPR/Cas9	74
	Low gluten wheat for reduced allergenicity Alpha-gliadin array	Wheat	CRISPR/Cas9	75
	Increased beta-carotene	Banana	CRISPR/Cas9	76
	Reductions of linoleic acid and linolenic acid	*Brassica napus*	CRISPR/Cas9	77
	Increased oleic acid content	*Camelina sativa*	CRISPR/Cas9	78

## 5. Biodiversity and environmental impact, and sustainable advance

If the needs of the world’s ever-growing population are to be met, global food production must be doubled by 2050 [79]. GE technologies’ good influence on yield is linked to a decrease in farming approaches in relation to water, energy, land, and agricultural chemicals, despite the relatively inelastic demand for food. One possible solution is to use GE to produce more high-quality food [80]. One advantage of using GE in agriculture is that it reduces the time required to achieve the desired trait or variety of plants, as well as pesticide use. We have already looked at the impact of GMOs on pesticide use; and the impact of GMOs on acreage utilization. According to Barrows et al., [72]2014, maintaining comparable productivity for soy, cotton, and corn without GE crops can necessitate at least an additional 13 million hectares of agriculture in 2010. As per other studies, a worldwide prohibition on GMOs results in a 3.1 million hectare increase in overall agriculture, with 0.6 million coming from forest degradation [81]. Relative to an extreme theory in which GMOs acceptance is raised to line with that of the United States, worldwide agriculture acreage shrinks by 0.8 million hectares [81]. Reducing agriculture’s land footprint, and the destruction of tropical forests, in particular, has a significant effect on climate change. Range from 3.1 million to 20 million hectares of additional cropland, with a consensus estimate of greenhouse gas emissions from the land cover change of 351 metric tons per hectare of changed soil 351 metric tons per hectare of transformed soil [82,83]. However, this is a one-time estimate rather than an annual total, such figures are similar to 19–135% of a year’s emissions in the US, which were 5.17 Gt in 2016 (U.S. EPA, 2012). Associated with reduced emissions from changes in land cover, the GE system can help with other elements of agricultural emissions, such as fossil fuel lowering and energy usage and allowing decreased spadework and cultivation. Weeding and chemicals have traditionally been used to keep undesired weeds from developing in the land. Herbicides are poisonous to most plants and must be used when the crop is not present. Herbicide-resistant plants enable chemical treatment while the crop is growing. The herbicide kills the weeds while leaving the GM crop untouched. The crop’s main herbicides required tillage to be successful due to the development of herbicide-resistant canola cultivars [84]. Herbicide treatment rates on canola in western Canada declined by 53% between 1995 and 2006 after the introduction of herbicide-resistant canola cultivars, but the number of farmers utilizing zero- or low-tillage increased to 64% [84]. The trend was found in the United States, where the acreage of soy under no-till management expanded by 65% between 1995 and 2009, the majority of growers cited herbicide-tolerant soy as the most important reason for switching to the no techniques [15,85]. Fuel consumption per acre was reduced by 11.8%, and overall greenhouse gas emissions decreased by 4.8 Mt, as a result of the move to no-till practices in soy production in the United States. The development of herbicide-tolerant crops resulted in a global shift to no-till techniques, resulting in the sequestration of 17.6 Mt of CO_2_ in the soil. Agricultural biodiversity, which is enabled by genetic engineering, provides for the protection of heirloom diversities that strength otherwise is missing, as well as crop biodiversity in general (Barrows et al., [71]2014). A survey-based study is carried out with 49 researchers from a regional bioeconomy research program in southern Germany [86]. The decreased cost of launching a feature into a particular crop trend driven by technologies like clustered regularly interspaced short palindromic repeats- genetic engineered (CRISPR-GE) does not indicate a loss in crop variety.

## 6. Risk and challenges of GMCs

Even though the existence of the gene moved is found obviously in other species, altering an organism’s normal state through the expression of alien genes has uncertain repercussions. Variations in metabolism, rate of growth, and reaction to external environmental stimuli appear to be influenced. Such changes have an impact on the GMO as well as the natural environment with which it is allowed to grow. People may be exposed to novel allergies as a result of genetically engineered foods and the transmission of antibiotic-resistant genes to gut flora. Horizontal gene transfer of pesticide, herbicideor antibiotic resistance to other species would not only endanger humans, but would also cause ecological imbalances by allowing previously harmless plants to spread unchecked, increasing pathogen transmission between plants and animals. It is still impossible to rule out the opportunity of horizontal gene transfer between GMOs and other organisms, the risk is typically viewed as low. Horizontal gene transfer is rare in nature, and it can’t be replicated in the lab without changing the target genome to make it more receptive [33]. In research of transgenic fish released into natural populations of the same species, the grave repercussions of vertical gene transfer between GMOs and their wild-type counterparts were demonstrated [87–90]. The improved mating abilities of the genetically modified fish resulted in a decrease in the survivability of their progenies.

The procedure sets and maintains suitable processes and metrics for regulating, managing, and controlling risks identified during risk assessment.

- As a result of the capacity to unify national governing backgrounds, suitable biosafety decision-making created on scientific hazard valuation is ensured.
- If successfully implemented, the protocol has the potential to enhance biotechnology discovery, development, technology transfer, and capacity building while also achieving the goals of sustainable agriculture, conservation, and equitable sharing of technology benefits.
- A first-come, first-served method in which early efforts are focused on quickly carrying any events in line with the guidelines. Adding more rules at this point will simply exacerbate the level of noncompliance currently present.
- Developers and users of agricultural biotechnology recognize their role in the protocol and contribute to effective building capacity.

The risk management procedure is another emphasis of the economic/political side of the GMOs biosafety issue.

CRISPR-Cas9 genome editing allows for the modification of any gene in any plant species. This allows for more rapid genetic modification than other techniques due to its simplicity, efficiency, low cost, and target genetic variants [91]. This could also be used to genetically modify previously unnoticed plants. The potential for crop breeding and the development of sustainable agriculture is incomparable [92].

## 7. Biotechnology’s contributions to long-term sustainability

Biotechnology describes a set of technological solutions which can be used in a wide range of industries [93,94]. Protein engineering, genetic engineering, and metabolic engineering are the three distinct branches of biotechnology. Biotechnology, particularly as it relates to living organisms, has been the subject of public debate. This should be noted that the issues of food safety and biosafety may be fundamentally opposed. A quarter correction termed variously as bioprocess, or biotechnology engineering, biochemical, is compulsory for profitable manufacturing of biotechnology foodstuffs and distribution of its service. None of the methods encircled by biotechnology are suitable throughout all industries [93]. Industries that had never given biological sciences a second thought as having an effect on the business are now looking into how they may use biotechnology to their advantage. Biotechnology offers completely new possibilities for long-term production. Environmental issues drive the industry to use biotechnology to not only eliminate contaminants but also to reduce contamination from arising. Biocatalyst-based processes play a significant role in this area. Microbial manufacturing processes are appealing because they make a varied assortment of molecules with low-energy processes using the elementary renewable resources of water, sunlight, and Co_2_. Such methods have been fine-tuned over time to enable a high-fidelity, efficient combination of less-toxicity compounds. Biotechnology has the potential to provide renewable bioenergy and is leading to the development of environment protection monitoring techniques. Biotechnology is previously been widely used, particularly in the production of biopharmaceuticals. Biotechnology is being used to create wholly new items in the adding to if innovative avenues. New economic areas such as nanobiotechnology and bioelectronics are emerging as a result of combining biotechnology with other growing fields [95]. Moreover, by conducting a scientific risk assessment and implementing preventive and corrective measures, the risks (contradictions) could be minimized or avoided kept to a minimum. Biotechnology has made a difference in sustainable farming in the accompanying directions.

- Enhanced biomass-derived energy generation innovations.
- Productivity and quality have risen.
- High nutrient levels can be generated in nutritionally crop varieties.
- Resistance to biotic stresses has been enhanced.

Biotechnology helps to ensure sustainable agriculture by putting limits on agrochemicals, particularly pesticides, through the use of genes that confer resistance to biotic and abiotic stresses. Genes carefully chosen from related or unrelated genetic resources are integrated into otherwise desirable genotypes. The systematic pyramiding of genes enables the introduction of desirable genes for different traits, like stress responses, efficiency, and nutritive value, into a single genotype.

## 8. Risk management techniques

Risk management and mitigation provide input that allows the initial evaluation to be validated. Threats can vary based on a number of circumstances, including the GMOs nature, intended application, and the environment in which it is released. Biotechnology is possibly the most well-known of the key new skills which are emerged since the 1970s. Biotechnology consumes proven effective in creating great wealth and influencing every major economic area. Biotechnology has already had a significant impact on environmental defense, healthcare, agriculture and forestry, food processing, and chemical manufacturing. Such assessment emphasizes biotechnology’s accomplishments and upcoming possibilities in the sustainable manufacture of goods and services, particularly those that are currently sourced often from the old chemical sector. As a result, they should be evaluated and managed on an individual basis. When applicable, the following facts must be added:

- Likelihood of endurance and permanence in the recipient ecosystem, as well as any potential selection advantage: If there is a selection benefit, the nature of it should be determined, as well as any potential negative consequences.
- The probability of gene transfer.
- Interactions with microbes have the potential to cause undesirable effects or consequences.
- Possible animal, human, and plant consequences.
- Potential biogeochemical process impacts or (nonreversible) disturbances.
- In general, possible dangers associated with the use of GMOs are reduced by hazard organization measures, which may type approximately planned actions suitable. It can be accomplished, through the use of confinement and monitoring measures.

## 9. Conclusions

This article discusses genetic modifications of industrial crops as well as the associated sustainability aspects for long-term development. The implementation and farming of GMC have made it the world’s fastest-growing agricultural technology. Using complementary new breeding techniques has the potential to provide solutions to food security and changing climate conditions, potentially introducing a broader range of more desirable food products. GMOs proponents suitable enough research for the commercialization of crop production. Guidelines on GMC cultivation vary greatly around the world, with some more mature in their experiences and thus more flexible to accommodate the entry of gene-edited products for approval. Genome editing and CRISPR-Cas9 in particular is a game-changing tool that has the potential to impact science, agricultural production, and the community. GMOs assist humanity when they are used to increase the availability and excellence of medical care and food, as well as contribute to a cleaner environment. These have the possibility to improve poverty and illness around the world if used intelligently, and If utilized correctly, they can benefit the economy without causing more harm than good. Furthermore, the full potential of GMOs cannot be reached until the dangers associated with each new GMOs are thoroughly investigated.
